# Central role for BRAF in cardiac hypertrophy: rethinking the pathological–physiological divide

**DOI:** 10.1042/CS20220776

**Published:** 2023-01-18

**Authors:** Raffaele Altara, George W. Booz

**Affiliations:** 1 Department of Pathology, School of Medicine, University of Mississippi Medical Center, Jackson, MS, U.S.A.; 2 Department of Anatomy and Embryology, Maastricht University, Maastricht, The Netherlands; 3 Department of Pharmacology and Toxicology, School of Medicine, University of Mississippi Medical Center, Jackson, MS, U.S.A.

**Keywords:** Cardiac remodeling, Extracellular signal-regulated MAP kinases, Fibrosis, Myocardial contractility, RAF kinases

## Abstract

The RAF/MEK/ERK1/2 signaling cascade has been implicated in pathological cardiac hypertrophy downstream of some Gq-coupled receptors. The RAF family of kinases consists of three isoforms (ARAF, BRAF, and CRAF) and until recently most studies on this signaling pathway in the heart have focused on RAF1 (CRAF). In a recent issue of *Clinical Science*, Alharbi et al. utilized an inducible cardiac myocyte targeted knockout mouse model to define the role of BRAF in pathological versus physiological hypertrophy using angiotensin II and phenylephrine (PE) infusion, respectively. They reported that loss of BRAF attenuated both pathological cardiac hypertrophy and interstitial fibrosis. BRAF knockout decreased cardiac function with PE in male mice and enhanced both interstitial and perivascular cardiac fibrosis but had no effect on hypertrophy. In contrast, loss of BRAF attenuated physiological hypertrophy in female mice but had no effect on fibrosis or contractility. These observations extend those previously made by this group assessing the consequences of expressing an inducible activating mutant of BRAF in the heart and the benefit of enhancing RAF/MEK/ERK1/2 signaling by exploiting the ‘RAF paradox’. Additional studies are needed to better define the role of BRAF under conditions reflective of chronic stress on the heart due to the biomechanical stimulation exerted by hypertension. In addition, the role of BRAF and its activation in overt heart failure remains to be established. Nevertheless, the new findings highlight the potential importance of additional signaling events, perhaps related to RAF1 or ERK1/2 activation, in shaping BRAF signaling in a sex- and context-dependent manner.

## Cardiac hypertrophy—physiological and pathological

**Figure 1:**
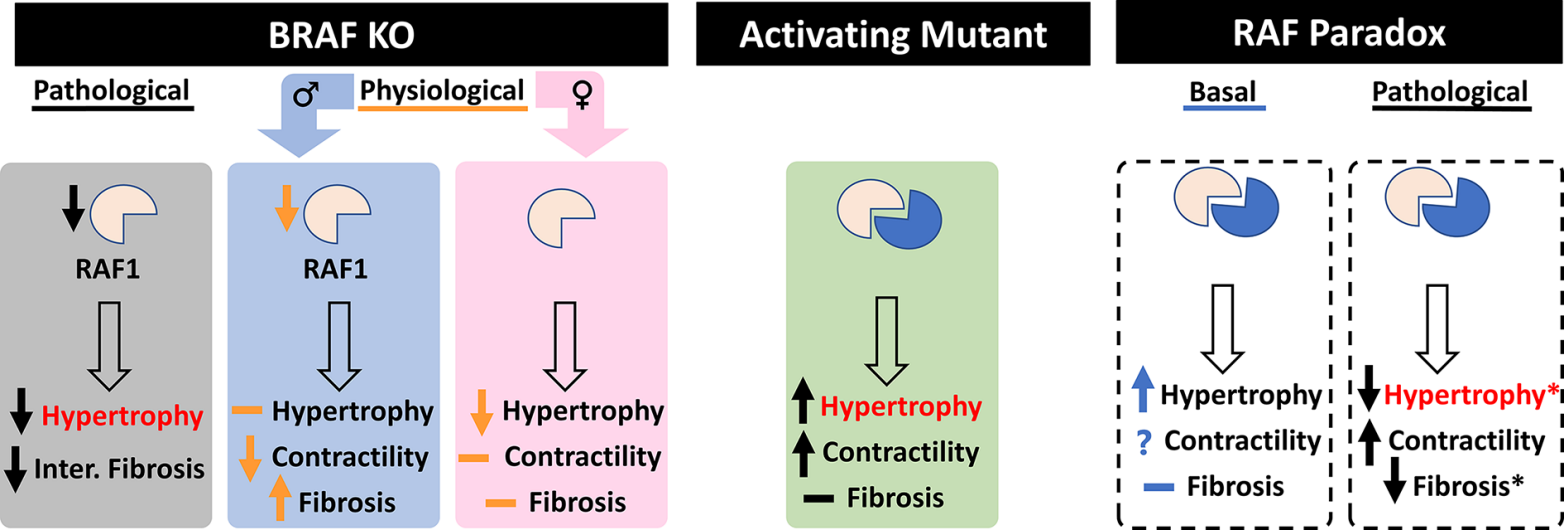
Summary of studies implicating BRAF in cardiac hypertrophy and fibrosis Heart targeted knockout studies have implicated BRAF in pathological hypertrophy and interstitial fibrosis. These studies were performed on male mice only and a sex difference was not studied. Divergent results were reported for the loss of BRAF on physiological hypertrophy in male versus female mice, but these results are complicated by a reduction in RAF1 in male mice only. Expression of a targeted knockin activated BRAF mutant was found to induce hypertrophy associated with an increase in markers of pathological hypertrophy, although contractility/cardiac function improved. Pharmacological activation of BRAF (the RAF paradox via Type 1 RAF inhibitors) had similar effects with the exception of pathological gene expression. Inexplicably, pharmacological activation of BRAF attenuated Ang II-induced pathological hypertrophy (as assessed by gene expression) and improved cardiac contractility/function. None of the gain-in-function studies for BRAF enhanced cardiac fibrosis, although loss of BRAF attenuated fibrosis under pathological conditions. Red text indicates pathological hypertrophy. The symbol ‘*’ indicates that only gene expression was reported. Contractility denotes contractility and/or cardiac function.

Increased workload on the heart induces cardiac myocyte growth in order to enhance cardiac contractility. The resultant increased cardiac muscle mass reduces elevated ventricular wall stress and compensates for increased hemodynamic demand. Depending upon the stimulus, the resultant cardiac hypertrophy may be classified as physiological or pathological [1]. Aerobic exercise and pregnancy cause physiological hypertrophy, whereas chronic hypertension and aortic stenosis cause pathological hypertrophy. Unlike physiological hypertrophy, pathological hypertrophy is associated with (interstitial) fibrosis, the re-expression of fetal genes, and capillary rarefaction. Overtime cardiac function declines with pathological hypertrophy and heart failure may result (Figure 1).

Multiple signaling pathways have been linked to pathological cardiac hypertrophy, most prominently two that are directly linked to increased intracellular calcium, calcineurin/nuclear factor of activated T-cells (NFAT), and Ca^2+^/calmodulin dependent kinase II (CamKII)/histone deacetylase (HDAC) [1,2]. Another prominent network for cardiac hypertrophy is the RAF/MEK/ERK1/2 (extracellular signal-regulated protein kinase 1/2) cascade, which may also be enhanced by elevated intracellular calcium. Biomechanical stress and neurohormonal factors can activate this signaling pathway and induce pathological cardiac hypertrophy [1]. G_q/11_-coupled receptors feed into RAF/MEK/ERK1/2 signaling with perhaps additional signaling input that regulates ERK1/2 nuclear localization [3].

## The Raf/MEK/ERK cascade

Activation of the RAF/MEK/ERK1/2 cascade occurs downstream of Ras small G-proteins and represents a major signaling pathway linked to G_q_-coupled receptors. Multiple lines of evidence have shown that ERK1/2 activation induces cardiac hypertrophy, yet also has a protective role under stress conditions [2,4]. Details on the activation of RAF kinases can be found elsewhere [5]; suffice it to say that homo- and heterodimerization is an important activation event. There are three RAF isoforms: ARAF, BRAF, and RAF1 (CRAF). Up until recently, most work in the heart dealing with this signaling cascade has focused on RAF1, which has been shown to be protective [2]. Yet BRAF has a higher basal activity than RAF1 and is more strongly activated by Ras than RAF1 [6]. One reason for the scarcity of studies on BRAF in the heart has been the lack of quality antibodies [7]. Recently, two studies originating from the same group have provided novel insights into the role of BRAF in cardiac hypertrophy. One of the studies appeared in *Clinical Science* and utilized an inducible cardiac myocyte-targeted BRAF knockout model. The second companion study involved a targeted knockin model of activated BRAF and the ‘RAF paradox’ activation by RAF Type 1 inhibitors. The present study also reported that cardiac BRAF and RAF1 (but not ARAF) mRNA and protein expression levels are up-regulated in patients with heart failure of mixed nonischemic etiology; although in patients with clearly defined dilated cardiomyopathy, BRAF is up-regulated while ARAF and RAF1 are down-regulated [7]. Together these studies revise our understanding of cardiac hypertrophy and raise the possibility that a class of drugs associated with cancer treatment might be repurposed to treat heart failure.

## BRAF knockout

Alharbi et al. [8] used an inducible knockout model to selectively eliminate BRAF in cardiac myocytes. These mice were subsequently infused using osmotic minipumps with either angiotensin II (Ang II) or PE to induce pathological or physiological cardiac hypertrophy, respectively. Although unsettled as far as clinical relevance, numerous studies have linked the Ang II type I (AT_1_) receptor to adverse cardiac remodeling [9]. PE also activates a G_q/11_-coupled receptor [3], the cardiac α_1A_-adrenergic receptor, which has a protective role in the heart [10].

Pathological hypertrophy was assessed in male mice, whereas both male and female mice were used for assessing physiological cardiac hypertrophy. Ang II induced cardiac myocyte hypertrophy as determined by measurement of cross-sectional area and echocardiography, as well as both interstitial and perivascular fibrosis. Knockout of BRAF inhibited cardiac hypertrophy and interstitial fibrosis but had no effect on perivascular fibrosis. Hypertrophic gene expression (*Nppa*, *Nppb*, but not *Myh7*) was attenuated as well. PE induced cardiac hypertrophy (as also assessed by an increase in heart-to-body weight ratio) and increased contractility, but not fibrosis, in male mice. This agonist did not increase mRNA expression of hypertrophic genes. In this case, knockout of BRAF had no effect on cardiac hypertrophy, whereas the increase in contractility was suppressed and both perivascular and interstitial fibrosis were enhanced. No explanation for the enhanced perivascular fibrosis is provided; however, cardiac blood vessels would be stimulated directly by the Ang II receptor and only subsequently the rest of the myocardium affected. Unlike in male mice, there was no decrease in RAF1 protein levels with BRAF knockout in female mice (see below). The PE-induced hypertrophic response of cardiac myocytes was modest in female mice and somewhat attenuated with BRAF knockout, although inexplicably the heart-to-body weight ratio was increased. Interstitial fibrosis with physiological cardiac hypertrophy was sporadic in female mice and not affected by BRAF knockout. There was no perivascular fibrosis with PE in female mice with or without BRAF knockout. No evidence was found for differential activation of ERK1/2 in male and female hearts of mice treated with PE in the absence or presence of BRAF, although their nuclear levels, related to ERK(Thr188) autophosphorylation and associated with pathological hypertrophy, were not assessed [11].

## Implications for cardiac remodeling

A salient feature of the study by Alharbi et al. [8] was the use of a slow pressor dose of Ang II that gradually increases blood pressure in mice over 7–14 days with a ‘limited’ effect at 7 days. Likewise, the dose of PE that was used would probably have increased blood pressure at 7 days by <10%. Thus, the growth effects on cardiac myocytes that were observed are likely attributable to agonist-induced receptor stimulation, rather than an overt effect of increased blood pressure. Although blood pressure was not monitored, historical support for blood pressure-independent effects for both Ang II and PE at the doses employed is provided. In addition, Ang II infusion did not increase the heart-to-body weight ratio at 7 days, a cardinal marker for cardiac hypertrophy at the gross level. However, others have reported that a comparable sub-pressor dose of Ang II did rapidly elevate blood pressure in mice when assessed with telemetry, although the increase declined to ∼10 mmHg in 7 days [12]. Moreover, PE stimulation may have pathophysiological consequences at higher doses [13]. For these reasons, it might perhaps be better to refer to the Ang II and PE models of Alharbi et al. [8] as ones of mild hypertension.

These results are to be contrasted with other studies that used a higher dose of Ang II that induces a rapid, sustained increase in blood pressure by 2 days, although the endpoint analyses were often done long after 7 days. Those studies generally reach the conclusion that Ang II does not have direct growth promoting effects on the heart and that biomechanical forces are predominate for causing cardiac hypertrophy [14]. A seminal study by Crowley et al. [15] using kidney transplants in mice reported that extrarenal AT_1A_ receptors are not needed for either hypertension or cardiac hypertrophy with infusion of a pressor dose of Ang II. Notably, the present study did not report echocardiographic data nor assessment of cardiac myocyte cross-sectional area but relied on the heart to body weight ratio as a measure of cardiac hypertrophy.

In the study of Alharbi et al. [8], Ang II promoted a ‘relatively small’ increase in interstitial fibrosis in localized areas over 7 days. Gene expression analysis implicated the involvement of profibrotic factors like fibroblast growth factor 2 (FGF2) and connective tissue growth factor (CTGF). The increase in their expression was eliminated by cardiac myocyte-targeted BRAF knockout. This finding supports the notion that cardiac fibrosis is driven by cardiac myocytes themselves, rather than being solely a direct effect of Ang II on cardiac fibroblasts [16]. In contrast, the study by Crowley et al. [15] did not extensively evaluate fibrosis in the heart, but did note that the hearts of kidney knockout mice infused with Ang II appeared ‘virtually’ normal. Their results suggest that fibrosis and vascular injury are both consequences of elevated blood pressure rather than a direct growth promoting effect of Ang II due to local actions involving cardiac AT_1_ receptors. Whether these results were influenced by possible suppression of the immune system after surgery is unclear [9].

A case could be made that a gradual increase in blood pressure as in the study of Alharbi et al. [8] better represents the real-world situation for the development of hypertension, rather than a sudden and abrupt increase observed either with a pressor dose of Ang II or with transverse aortic constriction (TAC). Alharbi et al. [8] propose that a subpressor dose of Ang II couples to cardiac remodeling via its action on arteriolar cells, such as smooth muscle or endothelial cells. In other words, Ang II may incite cardiac hypertrophy by intercellular signaling and paracrine factors involving the vasculature [17]. One possibility they suggest would be the production of endothelin-1 by endothelial cells, which has been demonstrated to have growth-promoting effects on cardiac myocytes [18]. Indeed, a recent study involving vascular ADAM17-deficient mice implicated VSMC ADAM17 in Ang II-induced cardiac hypertrophy, independent of increased blood pressure [19]. Furthermore, a recent study found that infusion of Ang II caused hypertrophy and hypertension in mice without AT_1_ receptors in the heart and conduit vessels, but not in mice lacking receptors in resistance vessels [20].

In contrast with Ang II, the hypertrophic effects of PE in the heart likely occur at the level of cardiac myocytes via α_1_-adrenergic receptor-mediated transactivation of insulin and insulin-like growth factor receptors with subsequent activation of phosphatidylinositol 3-kinase (PI3K) – protein kinase B (PKB) signaling, rather than via ERK1/2 activation [21]. This would lead to physiological hypertrophy [22,23]. Consistent with that, BRAF knockout did not significantly affect the increase in cardiac myocyte cross-sectional area seen with PE. In contrast, agonists that stimulate G_q_-coupled receptors, such as ET-1, would stimulate the BRAF/MEK/ERK cascade to induce pathological hypertrophy. Although BRAF is not involved in the growth promoting effects of PE on cardiac myocytes, this protein does appear to mediate some beneficial or cardioprotective actions of PE, such as enhanced contractility and inhibition of fibrosis. As suggested by the authors [8], both altered gene expression and nongenomic actions of ERK1/2 signaling may be involved. For instance, activation phosphorylation of NHE1 downstream of ERK1/2 may enhance contractility. Alternatively, these consequences of BRAF knockout might be attributable to a concomitant reduction in levels of RAF1 which is strongly cytoprotective [2,6]. The authors postulate that the reduction in RAF1 expression came about because RAF1 and BRAF form heterodimers so that with the loss of BRAF, RAF1 is not protected against degradation. The loss of RAF1 in the hearts of male mice following BRAF knockout might explain why cardiac fibrosis increased with PE, but was not increased in female mice as they did not show a similar loss.

## Exploiting the RAF paradox to obtain additional insights

In a companion study, Clerk et al. [7] assessed the consequences of expressing an inducible activating mutation of BRAF in the heart. These mice exhibited increased cardiac hypertrophy within 10 days as evidenced by echocardiography and increased cross-sectional area. In addition, the hearts exhibited enhanced cardiac function based on an increased ejection fraction and fractional shortening over 6 weeks, consistent with compensated hypertrophy. Although gene markers of pathological hypertrophy, fibrosis, and inflammation were increased, there was no cardiac fibrosis or evidence of cellular damage. This might be because the increases in mRNA were too modest or additional mechanisms of translational regulation were absent [7].

At non-saturating concentrations, Type 1 RAF inhibitors paradoxically enhance ERK1/2 signaling. An explanation for this phenomenon is that these inhibitors lock the other binding partner in the Raf dimer in an active conformation [7]. A pharmacological approach using two Type 1 RAF inhibitors at low concentrations, SB590885 and encorafenib, was taken by Clerk et al. [7] to further assess an apparent beneficial role for BRAF in the heart. Both of these agents, were shown to increase cardiac hypertrophy in mice as evidenced by increased cardiac myocyte size, with no effect on fibrosis. No effect was seen on *Nppa* or *Nppb* expression levels. Moreover, *in vitro*, both SB590885 and encorafenib increased ERK1/2 activity based on phosphorylation levels. It was also found that SB590885 increased nuclear ERK1/2 activity without affecting total nuclear levels, discounting the possibility that nuclear targeting of ERK1/2 is involved. *In vivo*, SB590885 was found to attenuate the decrease with subpressor Ang II in cardiac output, stroke volume, end diastolic volume, and end systolic volume, consistent with lessened hypertrophy and diastolic dysfunction. SB590885 modestly inhibited the Ang II-induced increases in the hypertrophic gene markers, *Nppa*, *Nppb*, and *Myh7*, but significantly blocked the reduction in *Myh6* expression. However, there was a marked inhibition of the AngII-induced increases in gene markers of fibrosis (*Ddr2*, *Col1a1*, *Fn1*, *Postn*) and inflammation (*IL1b* and *IL6*). Unfortunately, data directly assessing cardiac hypertrophy and fibrosis were not provided.

## Conclusions and future perspectives

Taken together, the findings by Clerk et al. [7] support the idea that BRAF signaling, and by extension the RAF/MEK/ERK1/2 cascade, is not necessarily pathological. Certainly, the outcome of BRAF activation may be dependent upon its level of activation. Moreover, a contribution of RAF1 to the activation phenotype of BRAF cannot be discounted. In addition to phosphorylating and inhibiting pro- or activating anti-apoptotic proteins [2,6,8], RAF1 might inhibit by some means profibrotic gene expression independent of MEK. This would explain the divergent effect of BRAF knockout on PE-induced cardiac fibrosis (males vs. females). The observation that BRAF knockout also reduced Ang II-induced cardiac fibrosis [8] in male mice is not easily explained but suggests that additional signaling events linked to G_q_ receptors, which shape the ERK1/2 response, are involved. Clerk et al. [7] suggests that ERK1/2 signaling in cardiac myocytes may be so hardwired as to be strictly compartmentalized, discounting the possibility that an increase in their translocation to the nucleus has a role. Additional investigation into this aspect of ERK1/2 signaling in the heart and its regulation are obviously needed.

It is important to note as well that while ERK1/2 signaling in the heart is pro-hypertrophic, evidence overall indicates that it is not obligatory [2]. Multiple other signaling molecules linked to Gq-coupled receptors that are also activated by biomechanical stress, including p38 and c-Jun N-terminal kinase (JNK) 1/2, have been implicated in pathologic cardiac hypertrophy [3,24]. In addition, ERK1/2 activation in the heart may fundamentally be protective and function to prevent the ventricular dilation that accompanies heart failure with reduced ejection fraction, while favoring concentric hypertrophy [25]. Nuclear targets of ERK1/2 that are linked to pathological hypertrophy and were associated with ERK1/2 Thr188 autophosphorylation, which is facilitated by the G_βγ_ complex, include mitogen- and stress-activated protein kinase 1 (MSK1), Elk1, and cMyc [26]. The molecular details of how autophosphorylation is regulated and whether Thr188 phosphorylation determines the actual profile of proteins targeted by ERK1/2 (beyond an effect attributable to increased nuclear levels) are not known [27]. A list of proteins targeted by ERK1/2 according to cellular localization may be found elsewhere [4].

The results of the studies by Clerk et al. [7] and Alharbi et al. [8] raise the possibility that Type 1 RAF inhibitors, used at a non-saturating concentration so as to enhance RAF activity, might have utility in treating or preventing heart failure by allowing for compensatory hypertrophy while blocking cardiac fibrosis. BRAF and MEK inhibitors when used together have proven highly successful in treating metastatic melanoma, although they are associated with cardiotoxicity [28]. As discussed by Clerk et al. [7], this is likely not to be a concern with the sole use of RAF inhibitors, although some (arguably manageable) risk of cancer might occur. However, both studies that have assessed the role of BRAF in ‘pathological’ hypertrophy have done so under condition of no or minimal effects on blood pressure. Thus, it would be prudent to investigate the importance of this kinase in the situation where blood pressure is chronically elevated, so that its contribution to biomechanical stress on the heart is clearly defined. In addition, none of the studies to date have actually assessed the role of BRAF and its activation in overt heart failure. In that context, a possible link of the beneficial effects of BRAF activation to RAF1 expression that was observed with physiological hypertrophy in male mice, may be a cautionary note.

## Data Availability

Data sharing is not applicable to the paper.

## Open Access

Open access for this article was enabled by the participation of University of Mississippi in an all-inclusive *Read & Publish* agreement with Portland Press and the Biochemical Society.

## Abbreviations

Ang II: angiotensin II
CTGF: connective tissue growth factor
FGF2: fibroblast growth factor 2
JNK: c-Jun N-terminal kinase
MSK1: mitogen- and stress-activated protein kinase 1
PI3K: phosphatidylinositol 3-kinase
PKB: protein kinase B
TAC: transverse aortic constriction
